# 2-(Acet­oxy­meth­yl)benzoic acid

**DOI:** 10.1107/S1600536813000780

**Published:** 2013-01-19

**Authors:** Graeme J. Gainsford, Ralf Schwörer

**Affiliations:** a Carbohydrate Chemistry Group, Industrial Research Limited, PO Box 31-310, Lower Hutt, New Zealand

## Abstract

The title compound, C_10_H_10_O_4_, crystallizes with the well-known carb­oxy­lic acid dimer-forming *R*_2_^2^(8) hydrogen-bond motif. Chains approximately parallel to (-1-12) are then built through C(methyl­ene,phen­yl)–H⋯O(carbon­yl) inter­actions [*C*(6) and *C*(8) motifs] with one (meth­yl)C—H⋯π inter­action providing inter­planar binding. The weakness of the latter inter­action is consistent with the difficulty experienced in obtaining suitable single crystals.

## Related literature

For details of the synthesis, see: Gorter-Laroij & Kooyman (1972 ▶). For related structures, see Kan *et al.* (2012 ▶); Liu *et al.* (2002 ▶); Valentine *et al.* (1992 ▶). For hydrogen-bonding motifs, see: Bernstein *et al.* (1995 ▶). For a description of the Cambridge Structural Database (CSD), see: Allen (2002 ▶).
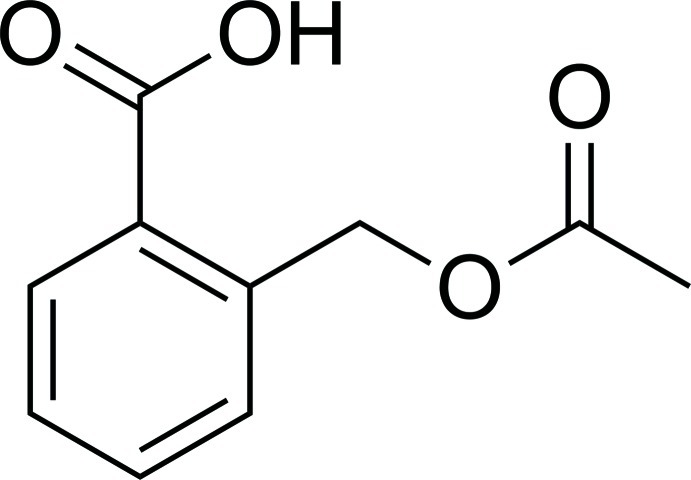


## Experimental

### 

#### Crystal data

C_10_H_10_O_4_
*M* *_r_* = 194.18
Triclinic, 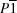

*a* = 6.2134 (9) Å
*b* = 8.2415 (9) Å
*c* = 9.6280 (11) Å
α = 77.54 (1)°
β = 83.364 (11)°
γ = 73.081 (12)°
*V* = 459.84 (10) Å^3^
*Z* = 2
Cu *K*α radiation
μ = 0.92 mm^−1^
*T* = 120 K
0.58 × 0.28 × 0.18 mm

#### Data collection

Oxford Diffraction SuperNova diffractometer
Absorption correction: multi-scan (*CrysAlis PRO*; Oxford Diffraction, 2007 ▶) *T*_min_ = 0.848, *T*_max_ = 1.000
2819 measured reflections
1767 independent reflections
1678 reflections with *I* > 2σ(*I*)
*R*_int_ = 0.012

#### Refinement

*R*[*F* ^2^ > 2σ(*F* ^2^)] = 0.033
*wR*(*F* ^2^) = 0.093
*S* = 1.08
1767 reflections
131 parameters
H atoms treated by a mixture of independent and constrained refinement
Δρ_max_ = 0.22 e Å^−3^
Δρ_min_ = −0.19 e Å^−3^

### 

Data collection: *CrysAlis PRO* (Oxford Diffraction, 2007 ▶); cell refinement: *CrysAlis PRO*; data reduction: *CrysAlis PRO*; program(s) used to solve structure: *SHELXS97* (Sheldrick, 2008 ▶); program(s) used to refine structure: *SHELXL97* (Sheldrick, 2008 ▶); molecular graphics: *ORTEP* in *WinGX* (Farrugia, 2012 ▶) and *Mercury* (Macrae *et al.*, 2008 ▶); software used to prepare material for publication: *SHELXL97* and *PLATON* (Spek, 2009 ▶).

## Supplementary Material

Crystal structure: contains datablock(s) global, I. DOI: 10.1107/S1600536813000780/fy2082sup1.cif

Structure factors: contains datablock(s) I. DOI: 10.1107/S1600536813000780/fy2082Isup2.hkl

Supplementary material file. DOI: 10.1107/S1600536813000780/fy2082Isup3.cml

Additional supplementary materials:  crystallographic information; 3D view; checkCIF report

## Figures and Tables

**Table 1:** Hydrogen-bond geometry (Å, °) *Cg*1 is the centroid of the C1–C6 phenyl ring.

*D*—H⋯*A*	*D*—H	H⋯*A*	*D*⋯*A*	*D*—H⋯*A*
O2—H2⋯O1^i^	0.971 (16)	1.667 (15)	2.6316 (12)	171.6 (12)
C4—H4⋯O4^ii^	0.95	2.43	3.3685 (15)	168
C8—H8*A*⋯O2^iii^	0.99	2.67	3.5747 (14)	152
C10—H10*B*⋯*Cg*1^iii^	0.98	2.82	3.5703 (13)	134

Symmetry codes: (i) 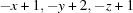; (ii) 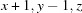; (iii) 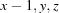.

**Fig. 1.**
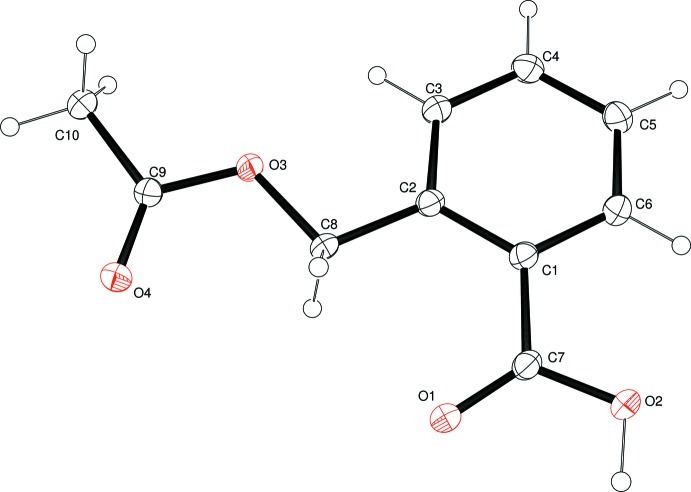
ORTEP view of the asymmetric unit with 30% ellipsoid probabilities. H atoms are of arbitrary size.

**Fig. 2.**
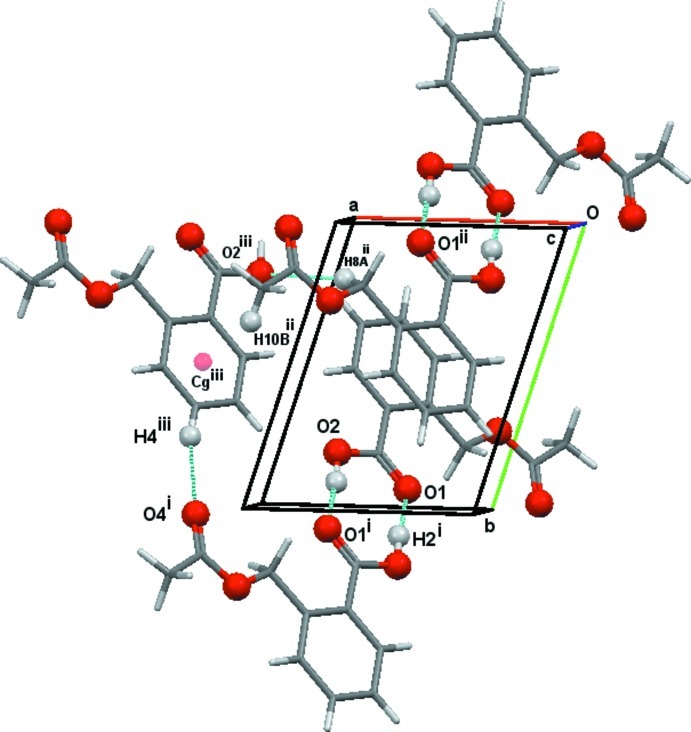
Cell contents view down the c axis. Contact atoms are shown as balls; intermolecular bonding contacts are shown as blue dotted lines. Symmetry: (i) 1 - x,2 - y, 1 - z (ii) 1 - x, 1 - y, 1 - z (iii) 2 - x, 1 - y, 1 - z.

## supplementary crystallographic information

### Comment

The title compound was synthesized during our studies on substituted benzoyl protecting groups that could be selectively cleaved in the presence of other benzoate esters. We believe the structure has not been reported previously because of difficulties, which we experienced, in obtaining suitable non-twinned single crystals and the tendency of the title compound to cyclize with formation of phthalide. The compound crystallizes with one independent C_10_H_10_O_4_ molecule in the asymmetric unit (Fig. 1). Only two closely related structures with similar carboxylic acid hydrogen bonding links [*R*^2^_2_(8) (Bernstein *et al.*,1995)] were found in the CSD (Allen, 2002): JOWTIY (Valentine *et al.*, 1992) and UHELOI (Liu *et al.*, 2002). The rather short intermolecular H2···O1 contact distance [1.667 (15) Å] (Table 1) is replicated in these two reports as 1.752 & 1.569 Å, respectively. A series of metal complexes containing the acetoxymethyl- moiety have been reported by Kan *et al.* (2012).

The crystal packing (Table 1) consists of the above-mentioned strong carboxylic acid hydrogen bonding in the plane of the molecule. This is coupled with C(methylene,phenyl)—H···O(carbonyl) interactions [C(6) & C(8) motifs] forming planar chains. One weak (methyl)C8—H10B···π interaction (labelled in Figure 2) crosslinks the planes of molecules, which are approximately parallel to the (-1,-1,2) crystal plane. This weak interplanar interaction is consistent with the difficulty in obtaining adequate non-twinned crystals.

### Experimental

The synthesis of the title compound has been reported previously by Gorter-Laroij & Kooyman (1972). Crystals for analysis were obtained by dissolving the title compound in a minimal amount of ethyl acetate, followed by addition of petroleum ether 60–80.

### Refinement

Eight outlier reflections, identified by large delta/sigma ratio (>4.8), were OMITted from the dataset (four were omitted on the basis of inconsistent equivalents). All methyl H atoms were constrained to an ideal geometry (C—H = 0.98 Å) with *U*_iso_(H) = 1.5*U*_eq_(C), but were allowed to rotate freely about the adjacent C—C bond. All other C bound H atoms were placed in geometrically idealized positions and constrained to ride on their parent atoms with C—H distances of 1.00 (primary), 0.99 (methylene) or 0.95 (phenyl) Å and with *U*_iso_(H) = 1.2*U*_eq_(C). The hydroxyl hydrogen on O2 was refined with *U*_iso_(H) = 1.2*U*_eq_(O2)

### Crystal data

**Table:**

C_10_H_10_O_4_	*Z* = 2
*M**_r_* = 194.18	*F*(000) = 204
Triclinic, *P*1	*D*_x_ = 1.402 Mg m^−^^3^
Hall symbol: -P 1	Cu *K*α radiation, λ = 1.54184 Å
*a* = 6.2134 (9) Å	Cell parameters from 1939 reflections
*b* = 8.2415 (9) Å	θ = 4.7–73.5°
*c* = 9.6280 (11) Å	µ = 0.92 mm^−^^1^
α = 77.54 (1)°	*T* = 120 K
β = 83.364 (11)°	Plate, colourless
γ = 73.081 (12)°	0.58 × 0.28 × 0.18 mm
*V* = 459.84 (10) Å^3^	

### Data collection

**Table:**

Oxford Diffraction SuperNova diffractometer	1767 independent reflections
Radiation source: fine-focus sealed tube	1678 reflections with *I* > 2σ(*I*)
Graphite monochromator	*R*_int_ = 0.012
Detector resolution: 10.6501 pixels mm^-1^	θ_max_ = 73.6°, θ_min_ = 8.1°
ω scans	*h* = −7→5
Absorption correction: multi-scan (*CrysAlis PRO*; Oxford Diffraction, 2007)	*k* = −10→9
*T*_min_ = 0.848, *T*_max_ = 1.000	*l* = −11→10
2819 measured reflections	

### Refinement

**Table:**

Refinement on *F*^2^	Primary atom site location: structure-invariant direct methods
Least-squares matrix: full	Secondary atom site location: difference Fourier map
*R*[*F*^2^ > 2σ(*F*^2^)] = 0.033	Hydrogen site location: inferred from neighbouring sites
*wR*(*F*^2^) = 0.093	H atoms treated by a mixture of independent and constrained refinement
*S* = 1.08	*w* = 1/[σ^2^(*F*_o_^2^) + (0.0557*P*)^2^ + 0.0727*P*] where *P* = (*F*_o_^2^ + 2*F*_c_^2^)/3
1767 reflections	(Δ/σ)_max_ < 0.001
131 parameters	Δρ_max_ = 0.22 e Å^−^^3^
0 restraints	Δρ_min_ = −0.19 e Å^−^^3^

### Special details

**Table:**

Geometry. All e.s.d.'s (except the e.s.d. in the dihedral angle between two l.s. planes) are estimated using the full covariance matrix. The cell e.s.d.'s are taken into account individually in the estimation of e.s.d.'s in distances, angles and torsion angles; correlations between e.s.d.'s in cell parameters are only used when they are defined by crystal symmetry. An approximate (isotropic) treatment of cell e.s.d.'s is used for estimating e.s.d.'s involving l.s. planes.
Refinement. Refinement of *F*^2^ against ALL reflections. The weighted *R*-factor *wR* and goodness of fit *S* are based on *F*^2^, conventional *R*-factors *R* are based on *F*, with *F* set to zero for negative *F*^2^. The threshold expression of *F*^2^ > σ(*F*^2^) is used only for calculating *R*-factors(gt) *etc*. and is not relevant to the choice of reflections for refinement. *R*-factors based on *F*^2^ are statistically about twice as large as those based on *F*, and *R*- factors based on ALL data will be even larger.

### Fractional atomic coordinates and isotropic or equivalent isotropic displacement parameters (Å^2^)

**Table:**

	*x*	*y*	*z*	*U*_iso_*/*U*_eq_	
O1	0.36320 (13)	0.93020 (10)	0.39619 (8)	0.0303 (2)	
O2	0.71012 (13)	0.80677 (11)	0.47419 (8)	0.0317 (2)	
H2	0.671 (2)	0.909 (2)	0.5167 (15)	0.038*	
O3	0.07354 (12)	0.72594 (9)	0.12998 (8)	0.0267 (2)	
O4	−0.20437 (13)	0.97266 (10)	0.12417 (8)	0.0313 (2)	
C1	0.56622 (17)	0.65287 (13)	0.34491 (11)	0.0249 (2)	
C2	0.40134 (17)	0.63516 (13)	0.26421 (10)	0.0239 (2)	
C3	0.44020 (18)	0.48029 (14)	0.21710 (11)	0.0285 (2)	
H3	0.3306	0.4661	0.1631	0.034*	
C4	0.6346 (2)	0.34570 (15)	0.24670 (13)	0.0325 (3)	
H4	0.6559	0.2413	0.2136	0.039*	
C5	0.79735 (19)	0.36433 (15)	0.32480 (12)	0.0330 (3)	
H5	0.9310	0.2733	0.3451	0.040*	
C6	0.76242 (19)	0.51722 (15)	0.37275 (12)	0.0307 (3)	
H6	0.8741	0.5304	0.4257	0.037*	
C7	0.53578 (17)	0.80933 (14)	0.40604 (11)	0.0260 (2)	
C8	0.18911 (17)	0.77882 (13)	0.22692 (11)	0.0251 (2)	
H8A	0.0919	0.7988	0.3140	0.030*	
H8B	0.2274	0.8874	0.1812	0.030*	
C9	−0.12281 (17)	0.83631 (13)	0.08618 (11)	0.0246 (2)	
C10	−0.22257 (18)	0.76614 (14)	−0.01435 (12)	0.0291 (3)	
H10A	−0.3748	0.8401	−0.0347	0.044*	
H10B	−0.2297	0.6487	0.0291	0.044*	
H10C	−0.1284	0.7637	−0.1033	0.044*	

### Atomic displacement parameters (Å^2^)

**Table:**

	*U*^11^	*U*^22^	*U*^33^	*U*^12^	*U*^13^	*U*^23^
O1	0.0287 (4)	0.0314 (4)	0.0345 (4)	−0.0057 (3)	−0.0068 (3)	−0.0149 (3)
O2	0.0268 (4)	0.0386 (5)	0.0348 (4)	−0.0073 (3)	−0.0075 (3)	−0.0172 (3)
O3	0.0254 (4)	0.0252 (4)	0.0323 (4)	−0.0038 (3)	−0.0085 (3)	−0.0120 (3)
O4	0.0306 (4)	0.0274 (4)	0.0362 (4)	−0.0014 (3)	−0.0064 (3)	−0.0129 (3)
C1	0.0251 (5)	0.0287 (5)	0.0217 (5)	−0.0074 (4)	−0.0017 (4)	−0.0066 (4)
C2	0.0247 (5)	0.0262 (5)	0.0218 (5)	−0.0079 (4)	−0.0011 (4)	−0.0060 (4)
C3	0.0294 (5)	0.0280 (5)	0.0306 (5)	−0.0073 (4)	−0.0049 (4)	−0.0098 (4)
C4	0.0351 (6)	0.0269 (5)	0.0347 (6)	−0.0035 (4)	−0.0035 (5)	−0.0104 (4)
C5	0.0296 (6)	0.0316 (6)	0.0329 (6)	0.0011 (4)	−0.0052 (4)	−0.0073 (4)
C6	0.0270 (5)	0.0376 (6)	0.0276 (5)	−0.0056 (4)	−0.0057 (4)	−0.0086 (4)
C7	0.0259 (5)	0.0325 (5)	0.0225 (5)	−0.0100 (4)	−0.0027 (4)	−0.0076 (4)
C8	0.0257 (5)	0.0262 (5)	0.0273 (5)	−0.0073 (4)	−0.0056 (4)	−0.0110 (4)
C9	0.0234 (5)	0.0260 (5)	0.0248 (5)	−0.0064 (4)	−0.0019 (4)	−0.0058 (4)
C10	0.0282 (5)	0.0317 (5)	0.0301 (5)	−0.0072 (4)	−0.0060 (4)	−0.0103 (4)

### Geometric parameters (Å, º)

**Table:**

O1—C7	1.2297 (14)	C3—H3	0.9500
O2—C7	1.3226 (12)	C4—C5	1.3885 (16)
O2—H2	0.974 (16)	C4—H4	0.9500
O3—C9	1.3421 (13)	C5—C6	1.3844 (17)
O3—C8	1.4454 (11)	C5—H5	0.9500
O4—C9	1.2068 (13)	C6—H6	0.9500
C1—C6	1.4003 (15)	C8—H8A	0.9900
C1—C2	1.4123 (14)	C8—H8B	0.9900
C1—C7	1.4848 (15)	C9—C10	1.4981 (14)
C2—C3	1.3922 (15)	C10—H10A	0.9800
C2—C8	1.5122 (14)	C10—H10B	0.9800
C3—C4	1.3905 (16)	C10—H10C	0.9800
			
C7—O2—H2	108.3 (8)	C1—C6—H6	119.3
C9—O3—C8	116.34 (8)	O1—C7—O2	122.58 (10)
C6—C1—C2	119.53 (10)	O1—C7—C1	123.34 (9)
C6—C1—C7	118.30 (10)	O2—C7—C1	114.07 (9)
C2—C1—C7	122.14 (9)	O3—C8—C2	107.39 (8)
C3—C2—C1	117.96 (10)	O3—C8—H8A	110.2
C3—C2—C8	119.97 (9)	C2—C8—H8A	110.2
C1—C2—C8	122.07 (9)	O3—C8—H8B	110.2
C4—C3—C2	122.02 (10)	C2—C8—H8B	110.2
C4—C3—H3	119.0	H8A—C8—H8B	108.5
C2—C3—H3	119.0	O4—C9—O3	123.58 (9)
C5—C4—C3	119.84 (10)	O4—C9—C10	125.78 (9)
C5—C4—H4	120.1	O3—C9—C10	110.65 (9)
C3—C4—H4	120.1	C9—C10—H10A	109.5
C6—C5—C4	119.18 (10)	C9—C10—H10B	109.5
C6—C5—H5	120.4	H10A—C10—H10B	109.5
C4—C5—H5	120.4	C9—C10—H10C	109.5
C5—C6—C1	121.47 (10)	H10A—C10—H10C	109.5
C5—C6—H6	119.3	H10B—C10—H10C	109.5
			
C6—C1—C2—C3	0.99 (15)	C7—C1—C6—C5	176.98 (10)
C7—C1—C2—C3	−176.96 (9)	C6—C1—C7—O1	−174.82 (10)
C6—C1—C2—C8	−178.20 (9)	C2—C1—C7—O1	3.15 (16)
C7—C1—C2—C8	3.86 (15)	C6—C1—C7—O2	4.31 (14)
C1—C2—C3—C4	−0.29 (16)	C2—C1—C7—O2	−177.72 (9)
C8—C2—C3—C4	178.91 (10)	C9—O3—C8—C2	178.87 (8)
C2—C3—C4—C5	−0.38 (17)	C3—C2—C8—O3	−6.19 (13)
C3—C4—C5—C6	0.34 (17)	C1—C2—C8—O3	172.98 (9)
C4—C5—C6—C1	0.37 (18)	C8—O3—C9—O4	−0.53 (14)
C2—C1—C6—C5	−1.05 (17)	C8—O3—C9—C10	179.49 (8)

### Hydrogen-bond geometry (Å, º)

Cg1 is the centroid of the C1–C6 phenyl ring.

**Table:**

*D*—H···*A*	*D*—H	H···*A*	*D*···*A*	*D*—H···*A*
O2—H2···O1^i^	0.971 (16)	1.667 (15)	2.6316 (12)	171.6 (12)
C4—H4···O4^ii^	0.95	2.43	3.3685 (15)	168
C8—H8*A*···O2^iii^	0.99	2.67	3.5747 (14)	152
C10—H10*B*···*Cg*1^iii^	0.98	2.82	3.5703 (13)	134

Symmetry codes: (i) −*x*+1, −*y*+2, −*z*+1; (ii) *x*+1, *y*−1, *z*; (iii) *x*−1, *y*, *z*.
